# Exploring the Relationship between Cognitive Ability Tilt and Job Performance

**DOI:** 10.3390/jintelligence11030044

**Published:** 2023-02-23

**Authors:** Anne E. Kato, Charles A. Scherbaum

**Affiliations:** 1 School of Business, Government, and Economics, Seattle Pacific University, Seattle, WA 98119, USA; 2 Department of Psychology, Baruch College, City University of New York, New York, NY 10010, USA

**Keywords:** specific abilities, ability tilt, cognitive ability/intelligence, GATB

## Abstract

Most of the work examining the relationship between intelligence and job performance has conceptualized intelligence as *g*. Recent findings, however, have supported the claim that more specific factors of intelligence contribute to the prediction of job performance. The present study builds upon prior work on specific cognitive abilities by investigating the relationship between ability tilt, a measure representing differential strength between two specific abilities, and job performance. It was hypothesized that ability tilt would differentially relate to job performance based on whether or not the tilt matched the ability requirements of the job, and that ability tilt would provide incremental validity over *g* and specific abilities for predicting performance when the tilt matched job requirements. Hypotheses were tested using a large sample from the General Aptitude Test Battery (GATB) database. Ability tilt related with job performance in the expected direction for 27 of the 36 tilt-job combinations examined, with a mean effect size of .04 when the tilt matched job requirements. The mean incremental validities for ability tilt were .007 over *g* and .003 over *g* and specific abilities, and, on average, tilt explained 7.1% of the total variance in job performance. The results provide limited evidence that ability tilt may be a useful predictor in addition to ability level, and contribute to our understanding of the role of specific abilities in the workplace.

## 1. Introduction

A century of research has produced considerable evidence that intelligence predicts job performance across multiple jobs and settings (e.g., Bobko et al. 1999; Hunter and Hunter 1984; Salgado et al. 2003; Sackett et al. 2022; Schmidt and Hunter 1998). Although originally focused on both general and specific cognitive abilities (e.g., Fleishman 1975; Fleishman et al. 1984), research gravitated to a position emphasizing that general cognitive ability (*g*) is the only ability from the cognitive domain that is needed to predict job performance (Ree et al. 1994, 2015; Schmidt and Hunter 1998; Schmidt 2002). However, recent work informed by modern cognitive ability theories and analytical techniques suggests that specific cognitive abilities contribute to the prediction of job performance and may, in some cases, offer greater predictive power than *g* (Hanges et al. 2015; Kell and Lang 2017, 2018; Lang et al. 2010; Mount et al. 2008; Nye et al. 2020, 2022; Reeve et al. 2015; Wee et al. 2014; Viswesvaran and Ones 2002; Wiernik et al. 2015).

This pivot away from “only *g*” toward once again recognizing the value of specific cognitive abilities creates a unique opportunity to draw on cognitive ability research from different fields, such as the research on ability tilt. Ability tilt refers to an intra-individual pattern of ability characterized by relative strength in one specific ability compared to another (Cattell 1987; Coyle et al. 2014). Most research to date has focused on tilts between quantitative and verbal abilities; more recent work has examined tilt between technical and academic abilities. The findings indicate that tilt predicts domain-specific academic and career outcomes commensurate with the pattern of the tilt. Quantitative tilt (math > verbal) predicts career outcomes in science, technology, engineering, and math (STEM) fields, while verbal tilt (verbal > math) predicts humanities and artistic outcomes (Coyle et al. 2014; Kell et al. 2013; Makel et al. 2016; Park et al. 2008; Wai et al. 2009, 2022). Similarly, academic tilt (academic > technical) predicts scores on college aptitude tests, whereas tech tilt (technical > academic) predicts STEM criteria (Coyle 2019, 2020). This work has also shown that ability tilt contributes unique predictive validity over *g* (Coyle et al. 2014). While ability tilt research has established links between tilt and distal career-related outcomes, very little research has examined the role of ability tilt for predicting job performance.

The present study builds upon existing research by examining the relationship between job-relevant cognitive ability tilt and job performance in a sample of working adults of varying ability levels. It was hypothesized that tilt would relate positively to job performance when the tilt pattern matches job requirements, and that tilt would relate negatively to job performance when the tilt pattern does not match job requirements. It was further hypothesized that ability tilt would provide incremental validity over *g* and specific abilities for predicting performance when the tilt matches job requirements.

### 1.1. Conceptualizations of Cognitive Ability

Cognitive ability involves the capacity to “reason, plan, solve problems, think abstractly, comprehend complex ideas, learn quickly and learn from experience” (Gottfredson 1997). Early research on the psychometric properties of cognitive ability focused on two opposing perspectives: Spearman’s (1904) two-factor model of intelligence, which emphasized a latent general factor (*g*), and Thurstone’s (1938) model of primary mental abilities, which emphasized the importance of more specific factors. These contrasting theoretical frameworks led to the development of the first hierarchical models of cognitive ability, in which various specific ability factors were grouped under *g* (see Brody 2000, for a historical review).

**Figure 1:**
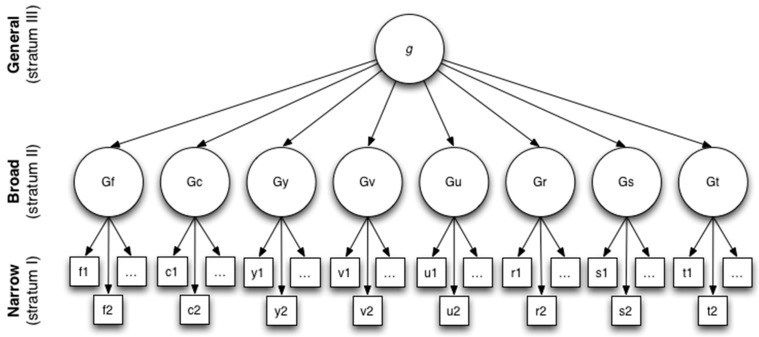
The Cattell-Horn-Carroll model of cognitive ability.

Cognitive ability is now recognized as a multidimensional, hierarchically organized construct with multiple levels of abilities ranging from general to specific (Horn and Blankson 2012; McGrew 2009; Schneider and Newman 2015). Although various frameworks for the structure of cognitive ability have been proposed, there is broad consensus and empirical support for multi-strata models of cognitive ability (e.g., Carroll 1993; Cattell and Horn 1978; Johnson and Bouchard 2005; McGrew 2009; Vernon 1950). A prominent example is the Cattell–Horn–Carroll (CHC) framework (McGrew 2009; Schneider and McGrew 2018; Schneider and Newman 2015), which organizes cognitive ability into three hierarchical tiers, as shown in Figure 1. CHC is a good exemplar of contemporary multi-strata models because it is compatible with multiple theories of cognition, permitting conceptual disagreements while providing a common nomenclature for the study of cognitive abilities. For example, the CHC framework welcome debates regarding the number of abilities that should be included in each stratum, and it takes an agnostic position on the nature of *g*, acknowledging that some view *g* as a higher-order psychological construct (Spearman 1904), while others interpret *g* as a psychometric phenomenon that emerges from positive interactions between cognitive processes (Lang et al. 2010; Van Der Maas et al. 2006). Overall, multi-strata models such as CHC promote the view that specific abilities are important for understanding human cognitive ability and are useful for identifying predictors of cognitive aspects of job performance (Horn and Blankson 2012; McGrew 2009; Schneider and Newman 2015).

### 1.2. Cognitive Ability and Job Performance

Several large meta-analytic studies have estimated the correlation between *g* and overall job performance (e.g., Bobko et al. 1999; Hunter and Hunter 1984; Sackett et al. 2022; Schmidt and Hunter 1998). The current estimate of the observed relationship is 0.24 (or .31 for the operational validity; Sackett et al. 2022). The accumulated evidence that *g* is an effective predictor, combined with a relative lack of evidence supporting the validity of more specific factors, has led some to conclude that “not much more than *g*” is required for predicting job performance (Ree et al. 1994, 2015; Schmidt 2002).

Recent work, however, has shown support for the claim that specific abilities may be useful for predicting job performance. For example, Lang et al. (2010) found that, when modern statistical techniques were used to assess the relative importance of general cognitive ability and specific cognitive abilities in predicting job performance, the importance of certain specific abilities (e.g., verbal comprehension, numerical facility, reasoning, and word fluency) was equal to or greater than that of *g*. The work of Wee et al. (2014) suggests that using Pareto-optimal weights of specific cognitive abilities may predict job performance at the same level of validity as *g*, while also reducing adverse impact. Nye et al. (2020) found that performance-based assessments of specific cognitive abilities added sizable incremental validity over *g* for predicting the training performance of Navy pilots. In addition to these primary studies, there is meta-analytic evidence that specific cognitive abilities show incremental validity over *g* for a variety of performance criteria, and the increments to validity are greatest for specific abilities that are less strongly correlated with *g* (e.g., visual processing, general knowledge, processing speed; Nye et al. 2022).

The above findings signify that a growing contingent of researchers are reconsidering the validity of specific abilities for cognitive ability assessment in the workplace (Hanges et al. 2015; Kell and Lang 2017, 2018; Nye et al. 2022; Oswald 2019; Reeve et al. 2015; Schneider and McGrew 2019; Wee et al. 2015; Wee 2018). This renewed interest in specific abilities in organizational research and practice creates opportunities to leverage modern cognitive ability research from other fields, such as the work on ability tilt.

### 1.3. Ability Tilt

The concept of ability tilt was introduced in the research of Lubinski and colleagues (Lubinski 2009; Lubinski et al. 2001; Park et al. 2008). They broadly defined ability tilt as an asymmetry between two specific abilities, and operationalized tilt as the within-person score difference between measures of two abilities. For example, ability tilt could be measured by subtracting an individual’s SAT verbal score from his or her SAT math score, and interpreting the resulting value (“tilt score”) as an indicator of either quantitative tilt (positive value) or verbal tilt (negative value). The absolute value of a tilt score indicates the magnitude of the tilt. A large tilt score suggests that one ability is considerably stronger than the other, whereas a small tilt score suggests that the two abilities are relatively balanced. According to this definition, ability tilt represents a pattern of cognitive ability that is conceptually distinct from ability level.

The following example illustrates the distinction between ability tilt and ability level. Consider two individuals who have taken the SAT. Both have a tilt score of 200, indicating that they have a quantitative tilt of the same magnitude. However, the first individual’s subtest scores are 800 on math and 600 on verbal, whereas the second individual’s subtest scores are 500 on math and 300 on verbal. Therefore, while both individuals exhibit the same degree of tilt toward the quantitative domain, their ability level differs considerably. On the other hand, consider two individuals who both have a total test score of 1400. The first individual’s subtest scores are 700 on math and 700 on verbal, whereas the second individual’s subtest scores are 800 on math and 600 on verbal. In this case, the test scores indicate that these two individuals have a comparable ability level and yet their specific ability pattern differs—one has equal ability across the two domains, whereas the other has a clear quantitative tilt. Thus, tilt provides insight into an individual’s relative strengths and weaknesses across domains.

The emergence of ability tilt in individuals has typically been explained in terms of investment theory. The investment model of intelligence (Cattell 1957) is based on the idea that the development of specific abilities results from domain-specific investment of cognitive effort. According to investment theory, cognitive development is influenced by various psychological and environmental factors, including an individual’s biology, interests, effort, motivation, and opportunities (Cattell 1987; Kvist and Gustafsson 2008). These factors may lead an individual to differentially invest across various domains. Investing effort in certain cognitive domains strengthens an individual’s ability in those domains while hindering the development of ability in competing domains, resulting in relative strengths and weaknesses across domains. Thus, over time, this process produces distinct patterns of cognitive ability within an individual, such as ability tilt, which may have implications for the development of expertise in various domains.

Research has established that ability tilt is useful for predicting outcomes within specific domains related to the tilt pattern. The earliest findings originated from Lubinski and colleagues’ 25-year longitudinal study of intellectually gifted adolescents who took the SAT by age 13 (Kell et al. 2013; Lubinski et al. 2001; Lubinski 2009; Park et al. 2008). They found that quantitative tilt predicted achievements in STEM (e.g., STEM degrees and patents) while verbal tilt predicted achievements in the humanities (e.g., humanities degrees and literary publications). That is, while all individuals in this high ability sample demonstrated high achievement, tilt was useful for predicting the content or domain of their achievements. Additionally, tilt scores were unrelated to total SAT scores (Park et al. 2008), which have been found to correlate strongly with *g* (Coyle and Pillow 2008). These findings suggest that ability tilt offers unique insight into the prediction of future accomplishments beyond an overall level of achievement, and that tilt effects are not merely a function of *g*.

In addition to research examining tilt in highly gifted individuals, other research has found similar effects with more representative samples. For example, Wai and colleagues (2009) found that postsecondary degrees and occupations varied based on ability tilt patterns identified from a wide range of tests. The tests were administered to a stratified random sample of U.S. high school students and tracked for 11 years after graduation. They found similar patterns in a sample of Graduate Record Examination (GRE) test-takers. These results were replicated in subsequent research, where some differences were found between the GRE sample and the sample drawn from the general population, and yet the overall pattern of results was consistent (Wai et al. 2022).

Coyle and colleagues have also examined relations between ability tilt and a variety of outcomes using data from the National Longitudinal Survey of Youth comprising scores from the SAT, ACT, and Armed Services Vocational Aptitude Battery (ASVAB). They found that ability tilt differentially predicted college major, occupational preferences, and other domain-specific outcomes (Coyle et al. 2014; Coyle et al. 2015; Coyle 2016, 2019, 2020). Quantitative tilt was associated with STEM majors, whereas verbal tilt was associated with humanities majors. Similarly, tech tilt (technical > math or verbal) was associated with majors and jobs in STEM, whereas academic tilt (math or verbal > technical) was associated with majors and jobs in humanities (with the exception of technical > math tilt, which correlated near zero with college major). Each type of tilt was also found to be negatively related to majors in competing fields.

In sum, the literature suggests that cognitive ability tilt is best understood as an individual’s relative pattern of strength and weakness in one specific cognitive ability over another. Whereas *g* or specific abilities reflect an individual’s level of ability, ability tilt provides insight into an individual’s ability pattern between domains. Empirical research on ability tilt has established that tilt predicts a variety of criteria that are relevant to workplace applications, such as occupational attraction, career choice, and domain-specific career outcomes. Nevertheless, little research has considered whether ability tilt may influence individuals’ job performance, or whether tilt scores may be useful for predicting job performance. Additionally, no research has explicitly examined tilt in a representative sample of working adults. The present study investigates these issues.

### 1.4. Ability Tilt and Job Performance

Predicting job performance is predicated upon a thorough understanding of job tasks, as well as the human attributes (i.e., KSAOs) that are necessary to perform them. In other words, job requirements provide an important basis for choosing predictors. Cognitive ability is necessary for many jobs; however, jobs vary considerably in terms of the specific cognitive abilities they require (Fleishman et al. 1999). It should not be surprising, then, that the criterion-related validity of specific cognitive abilities varies across different jobs (Johnson et al. 2010; Steel and Kammeyer-Mueller 2009). While little empirical research has examined relations between specific abilities and performance in specific jobs, theory suggests that specific abilities will be most predictive of performance in jobs that explicitly require them (Schneider and Newman 2015). Recent work has also suggested the importance of matching specific cognitive abilities to job tasks for the predictive validity of specific abilities (Krumm et al. 2014).

This line of reasoning can be extended to the different patterns of cognitive ability that are required by different jobs. Jobs vary in the number of specific cognitive abilities that are required for performance and the level of each ability required. For example, verbal ability is paramount for the job of an English teacher, whereas quantitative, spatial, and verbal abilities are all equally necessary for the job of a civil engineer. Put another way, different jobs require different types and magnitudes of tilt between specific abilities. Thus, ability tilt should be beneficial when the tilt pattern corresponds to job requirements. This view is consistent with Connell et al.’s (2003) framework of cognitive expertise, which suggests that individuals are most likely to perform well when their ability profiles match the requirements of the job. It is also consistent with the demands–abilities conceptualization of person–job fit, which is defined as the correspondence between an employee’s KSAOs and job demands (Edwards 1991) and relates positively with job performance (Kristof-Brown et al. 2005). These frameworks suggest a motivational effect of tilt, wherein employees are most motivated to acquire job-relevant knowledge and expend effort in jobs that capitalize on their strengths and are demotivated in jobs that emphasize their weaknesses. Just as prior ability tilt research has found that individuals are more likely to demonstrate interest and accomplishments in domains that reflect the relative strength represented by their tilt, person–job fit research suggests tilt may predict job performance based on how well a person’s ability tilt matches job requirements. Thus, it is expected that the relationship between ability tilt and job performance will vary based on the correspondence between the tilt and the specific ability requirements of the job. The present study examines relations between ability tilt and job performance under the two conditions of a match and a mismatch between the tilt and job requirements.

Additionally, it is proposed that tilt will provide incremental validity over measures of ability level (i.e., *g* and specific cognitive abilities) for predicting job performance. Research has demonstrated that the predictive validity of cognitive ability can be improved when job-relevant specific abilities are used in conjunction with *g* (Nye et al. 2020). Ability tilt offers a means of further optimizing the fit between an individual’s cognitive abilities and job demands. Theoretically, there is no reason to expect that having a relative strength in one ability over another can substitute for having the requisite level of cognitive ability; however, given two individuals with relatively equal ability, the individual with the specific ability pattern that more closely matches job requirements should perform better. Thus, the key question is whether tilt offers incremental validity for predicting performance in jobs that require relative strength in one cognitive ability over another. Based on prior ability tilt research and the frameworks of cognitive expertise and demands–abilities fit outlined above, it is expected that ability tilt will provide incremental validity beyond *g* and specific abilities for predicting job performance when the tilt matches job requirements.

**Hypothesis** **1a.** *Ability tilt is positively related to job performance when the tilt matches job requirements, when controlling for g.*

**Hypothesis** **1b.** *Ability tilt is negatively related to job performance when the tilt does not match job requirements, when controlling for g.*

**Hypothesis** **2.** *Ability tilt explains unique variance in job performance beyond g when the tilt matches job requirements.*

**Hypothesis** **3.** *Ability tilt explains unique variance in job performance beyond g and individual specific abilities when the tilt matches job requirements.*

In addition to examining relations between ability tilt and performance in jobs with tilted ability requirements, we were also interested in understanding the relationship between ability tilt and job performance in jobs with balanced ability requirements (i.e., jobs that require multiple specific cognitive abilities to approximately equal degrees). Based on cognitive expertise theory (Connell et al. 2003), we suspected that ability tilt would relate negatively to performance in these “generalist” jobs because stronger tilts would prevent a balanced application of several specific abilities as required by the job. However, due to a lack of previous empirical findings on which to base a hypothesis, these relationships were examined as a research question.

## 2. Method

### 2.1. Sample/Data

This study utilized data from the General Aptitude Test Battery (GATB). The GATB consists of 12 ability tests (eight paper-and-pencil tests plus four apparatus tests) that measure nine cognitive aptitudes, as shown in Table 1 (U.S. Department of Labor 1970). Although the GATB was developed based on factor analyses rather than a specific theory or model of cognitive ability, most of the GATB aptitudes are comparable to specific ability factors included in contemporary multi-strata models of cognitive ability such as CHC.

This study utilized a dataset comprising 40,489 individuals drawn from occupational settings who were administered the GATB (see Appendix A for more information). Available data included GATB scores, job performance criteria, and job information. All records were retained for participants age 18 years and older that used a standard supervisory rating scale as the job performance criterion, had no missing data for any of the study variables, and for which job analysis ratings for an equivalent job title were available in O*NET. This screening yielded a sample of 23,994 individuals from 80 jobs within 14 job families. Demographic characteristics of the sample are presented in Table 2.

### 2.2. Variables

#### 2.2.1. GATB Scores

GATB score data included standardized aptitude scores for each of the nine aptitudes. Scores on five of these aptitudes (V, N, S, P, Q) were examined. The rationale for excluding the other four aptitudes is that G is interpreted as a general ability factor (specific to the GATB, not equivalent to *g*) rather than a specific ability, and K, F, and M are psychomotor abilities. Although most versions of the CHC framework include psychomotor abilities (Gp) at the Stratum II level, this domain has received little attention in the literature and its factor structure is less well understood (Schneider and McGrew 2018). For parsimony, we chose to focus on specific abilities that have been examined in prior research.

#### 2.2.2. *g* Scores

*g* scores were estimated based on a principal components factor analysis of the nine GATB scales, consistent with CHC theory and prior research using GATB data. Following the approach taken by McDaniel and Kepes (2014), variables were loaded on the first factor to weight the scales, and the resulting factor scores were used as a measure of *g*. Specifically, *g* was defined as shown in the following equation:

*g* = G*.88974 + N*.83586 + P*.81291 + Q*.79446 + V*.78410 + S*.70622 + K*.59619 + F*.57248 + M*.50031 (1)

#### 2.2.3. Ability Tilt Scores

Tilt scores were computed as the within-person difference between two GATB aptitude scores. Tilt scores were computed in both directions such that all types of tilt are reported as positive values. For example, examination of V > N tilt was computed as V—N whereas N > V tilt was computed as N—V. The full range of tilt scores for each specific ability pair was utilized for analyses, except as noted.

The five GATB dimensions used for this study yield ten pairs of aptitudes and twenty types of tilt (see Table 3). However, tilts between form perception and clerical perception (i.e., P > Q and Q > P) were excluded from this study due to a lack of conceptual distinction between these two abilities in the corresponding O*NET ratings. Both P and Q are encompassed by the ability labeled “perceptual speed” in O*NET, and thus it was not possible to create contrasting job groups for these two abilities. P and Q were analyzed separately in the context of other tilt relationships according to the GATB conceptualization.

Prior ability tilt research has considered only two specific abilities at a time, and has thus used simple labels such as “verbal tilt” or “quantitative tilt” to describe a particular tilt pattern. This study is unique in examining tilts across five specific abilities. Given the number of variables included in this study, a general label such as “verbal tilt” could refer to four distinct types of tilt (see Table 3). Therefore, the labels used to denote tilt in this study make explicit reference to the two abilities involved in the tilt relationship (e.g., V > N tilt, N > V tilt, etc.). Usage of the more general tilt labels is limited to situations in which a comparison across related types of tilt is intended.

#### 2.2.4. Job Performance Criteria

Job performance criteria were supervisory ratings of overall job performance, taken from the standard descriptive rating scale used for USES research. Ratings were on a 5-point scale ranging from “definitely unsatisfactory” (1) to “outstanding” (5).

#### 2.2.5. Job Tilt Scores

Jobs in the sample were defined by codes from the *Dictionary of Occupational Titles* (DOT; U.S. Employment Service 1977). DOT codes were translated into the corresponding O*NET-SOC occupation codes. Job analysis ratings from O*NET were then used to calculate job tilt scores, as follows.

O*NET rates the ability requirements of various jobs using 21 specific cognitive abilities based on Fleishman’s taxonomy (Peterson et al. 2001). The abilities that correspond to the aptitudes measured by the GATB include: written comprehension, mathematical reasoning, number facility, visualization, and perceptual speed. Table 4 shows how these abilities align with the comparable GATB aptitudes. Because the GATB definition of numerical aptitude encompasses both mathematical reasoning and number facility, scores on these two abilities were combined into a composite variable labeled quantitative reasoning. This approach was justified given that the correlation between O*NET ratings of mathematical reasoning and number facility was .91 across all jobs in the sample.

O*NET job analysis data include ratings of ability importance and ability level. Importance ratings indicate an ability’s degree of importance to a job and are made on a 5-point scale ranging from “Not Important” (1) to “Extremely Important” (5) and mean ratings are standardized to a 0–100 scale. Level ratings indicate the degree to which the ability is required to perform a job, as specified by a point along a continuum ranging from 0–7. Mean ratings of both importance and level are standardized to a 0–100 scale for comparability. Because understanding of a job’s requirements is enhanced when the two ratings are considered together, both importance and level ratings were used to determine job requirements and job tilt scores.

First, the ability requirements for each job were computed using a unit-weighted combination of the importance and level scales, consistent with the approach taken for the most recent version of the O*NET occupational ability profiles (Allen et al. 2011). Second, job tilt scores were calculated by taking the difference between two specific ability requirements. For example, the job tilt score representing the difference between the written comprehension (WC) and visualization (Vz) abilities required for a job is represented as:

(Importance_WC_ + Level_WC_)—(Importance_Vz_ + Level_Vz_) (2)

#### 2.2.6. Job Groups

Job tilt scores were used to create job groups for this study’s analyses. Because job tilt scores varied considerably between the nine pairs of specific abilities examined in this study, sample parameters were used to determine tilted job groups rather than setting an absolute cutoff score across all types of tilt. Matched job groups (H1a) were defined as all jobs with job tilt scores greater than one standard deviation above the mean for each type of tilt. There were, however, two types of tilt for which no jobs met the one standard deviation criterion. Specifically, only one job in the sample required quantitative reasoning ability to a greater degree than written comprehension (QR > WC tilt), and only six jobs required quantitative reasoning ability to a greater degree than perceptual speed (QR > PS tilt). Thus, these job groups included all jobs with tilt scores greater than zero. Table 5 presents the sample sizes and sample job titles for each of the 18 job groups.

Mismatched job groups (H1b) were defined as all jobs with negative job tilt scores for each type of tilt (i.e., all jobs with requirements that tilted in the opposite direction of the ability tilt). This approach was based on the assumption that ability tilt would be beneficial only in jobs that clearly require the same type of specialization in one specific ability over another, not in jobs that require the opposite tilt pattern (including relatively “balanced” jobs with only a slight tilt in the opposite direction).

Jobs with balanced ability requirements (RQ1) were defined as the nine jobs with the smallest variance amongst their specific ability requirements (i.e., amongst requirements for written comprehension, quantitative reasoning, visualization, and perceptual speed).

## 3. Results

Table 6 presents the mean levels of ability tilt across all individuals in the sample. The first hypothesis predicted that ability tilt would relate positively to job performance when the tilt matches job requirements (H1a) and would relate negatively to job performance when the tilt does not match job requirements (H1b) after controlling for *g*. To control for *g*, analyses utilized standardized residuals computed from regressing *g* on each type of tilt in lieu of raw tilt scores.

Table 7 and Table 8 present the correlations between ability tilt and job performance for the 18 types of tilt included in this study, within the two conditions of matching and mismatched job requirements. In cases when ability tilt matched job requirements, 14 of the 18 types of ability tilt related positively to job performance as expected, of which 10 correlations were statistically significant. Four types of ability tilt related negatively to performance despite the match between tilt and job requirements, but only one was statistically significant. Specifically, in jobs that supposedly required more spatial than quantitative ability, S > N tilt was negatively related to job performance. Subsequent analyses at the individual job level revealed that one job, structural iron and steel workers, predominantly accounted for this counterintuitive effect. When this job was removed from the job group, the effect size decreased (*r* = −.023) and no longer met the threshold for statistical significance. Overall, the magnitude of the effects ranged from −.03 to .12 and the mean effect size was .04. Among the positive tilt-performance relationships, the mean effect size was .06 (.07 when restricted to statistically significant effects). Thus, results of these analyses partially support Hypothesis 1a.

When ability tilt was mismatched with job requirements, 13 of the 18 types of ability tilt related negatively to job performance as expected, of which 11 were statistically significant. Five types of ability tilt related positively to performance despite the mismatch between the tilt and job requirements, and four of these effects were statistically significant. All of these counterintuitive effects were instances in which numerical aptitude was the stronger of the two abilities in the tilt. In other words, quantitative ability tilts related positively with performance, even when they did not match the requirements of the job. Further analysis revealed that there were very few jobs in the sample that had quantitatively tilted requirements, so the mismatched job groups for these ability tilts comprised the majority of the jobs in the sample. Additionally, when the threshold for the mismatch was set higher, so that there was a clearer mismatch between the ability tilt and job requirements, the effects changed signs and/or became nonsignificant. Overall, the magnitude of the effects ranged from −.08 to .07 and the mean effect size was −.02. Among the negative tilt-performance relationships expected for the mismatched condition, the mean effect size was −.05. These results partially support Hypothesis 1b.

We predicted that ability tilt would explain unique variance in job performance beyond *g* alone (H2), as well as beyond a combination of *g* and job-relevant specific abilities (H3), in jobs with ability requirements that match the tilt pattern. Relative importance analyses were employed to test these hypotheses. The relative importance of a predictor is defined as “the proportionate contribution each predictor makes to *R*^2^, considering both its direct effect (i.e., its correlation with the criterion) and its effect when combined with the other variables in the regression equation” (Johnson and LeBreton 2004, p. 240). The relative weights (Johnson 2000) approach to determining relative importance was used in this study. Relative weights were computed for each job group using a model encompassing *g*, the dominant specific ability in the job tilt, and the job-relevant ability tilt.

Table 9 summarizes the results of the relative weight and incremental validity analyses. Relative weights for ability tilt ranged from 2.8% to 17.7% with a mean value of 7.1%. The values reflect the proportion of *R*^2^ that belongs to ability tilt. *R*^2^ values ranged from .027 to .07 which is consistent with prior research using the GATB data (e.g., McDaniel and Kepes 2014). Incremental validities for each model compared to a *g*-only model, as well as a model including both *g* and the dominant specific ability, are presented in the last column of Table 9. The incremental validity of ability tilt over *g* ranged from .000 to .017 with a mean of .007. Given the moderate effect sizes for *g* in these samples, these values represent percentage increases in *R*^2^ ranging from 0% to 43% (mean 13%) over *g* alone. The incremental validity of ability tilt over *g* plus job-relevant individual specific abilities ranged from .000 to .008 with a mean of .003, corresponding to percentage increases in *R*^2^ ranging from 0% to 15% (mean 5%). These results lend partial support for the hypothesis that ability tilt explains unique variance in job performance beyond *g* (H2) and limited support for the hypothesis that ability tilt explains unique variance beyond a combination of *g* and job-relevant specific abilities (H3).

## 4. Discussion

This study aimed to extend the literature on ability tilt by examining the relationship between cognitive ability tilt and job performance, and, in doing so, to explore a new application of specific cognitive abilities within the context of predicting job performance. Additionally, this study was the first to examine tilt effects in a sample of working adults representative of the general population, and did so across a broader range of specific abilities than has been studied in prior tilt research. The findings of this study contribute to our understanding of the relationship between specific cognitive abilities and job performance in several ways.

First, the results of this study suggest that the relationship between cognitive ability tilt and job performance depends on the requirements of the job. It appears that ability tilts that aligns with job requirements are, more often than not, beneficial for job performance, and ability tilts that diverge from job requirements are, more often than not, detrimental to performance. The finding that quantitative ability tilts were positively related to job performance, even in jobs that supposedly required the opposite pattern of specialization, raises questions about whether all ability tilts operate uniformly. These results would appear to suggest that quantitative ability has a disproportionate impact on job performance in comparison to job requirements, that is, it pays to specialize in quantitative ability regardless of the fit with the job. However, some caution should be exercised in interpreting these results, since other studies have found that verbal ability is a relatively more important predictor of performance compared to quantitative ability, which aligns with what O*NET job requirements suggest (Lang et al. 2010; Wee 2018).

It is possible that the counterintuitive quantitative tilt effects are attributable to idiosyncrasies of the sample. Although the GATB sample utilized for this study is believed to be representative of U.S. jobs, and has been used as the basis of much of the validity generalization research supporting the relationship between cognitive ability and job performance, the majority of jobs in this sample were low-complexity jobs such as production, administrative support, and installation and maintenance. Perhaps in these jobs, quantitative ability positively differentiates employees even when it is relatively less important to the job than another ability, especially when the job tilt is small. Another possibility is that the importance and level of quantitative ability required by various jobs is systematically underestimated in O*NET. While this may seem unlikely, a closer inspection of O*NET job analysis data revealed that quantitatively tilted job requirements were relatively rare across all jobs. For example, only 19 (2%) of the 966 jobs in O*NET supposedly require more quantitative than verbal ability. Other types of quantitative job tilts were more common, but still topped out at approximately one third of all jobs. Overall, these results point to the complexity of human intelligence, and suggest there is more to be learned regarding how specific abilities relate to performance in different jobs. More research with a wider range of jobs is needed to determine whether the relationships between quantitative ability tilts and job performance found in this study generalize to other samples, and, if so, why quantitative tilts are so valuable.

While relations between ability tilt and job performance generally displayed the expected pattern of results, based on effect sizes one could conclude that this study provided limited evidence for the utility of tilt as a predictor of job performance. When considering tilt along with measures of ability level (i.e., *g* and specific cognitive abilities), relative weights and incremental validities for tilt scores were small (see Table 9). However, contextualizing these small effect sizes provides a better understanding of the importance of tilt (Prentice and Miller 1992). In their seminal meta-analysis of the utility of various selection methods, Schmidt and Hunter (1998) define the utility of a predictor in terms of its percentage increase in validity over *g* alone. In this sample, the *R*^2^ for the relation between *g* and job performance across all jobs was .049, a value that is consistent with prior research using the GATB (e.g., McDaniel and Kepes 2014) and with revised meta-analytic estimates of the validity of cognitive ability (Sackett et al. 2022). Thus, a mean incremental *R*^2^ of .007 for ability tilt over *g* represents a 13% improvement in the total amount of criterion variance explained. For some tilted job groups, the improvement in prediction was considerably higher. However, the unique contribution of tilt was much lower when job-relevant specific abilities were included in the predictive model, averaging a 5% increase in criterion variance explained. Thus, the findings of the present study suggest that ability tilt provides unique insight into job performance in comparison to general cognitive ability, but may not contribute much beyond individual specific abilities.

Finally, the findings of this study suggest that the relationship between ability tilt and job performance may be complex in jobs that require a balanced profile of specific abilities. Whereas ability tilt is generally detrimental when there is a clear mismatch between the tilt and job requirements, other factors may influence the effect tilt has on performance in jobs with more balanced requirements. Perhaps some employees find ways to leverage their cognitive strengths or expertise in jobs that do not require it, whereas others rely too heavily on their dominant ability to the detriment of their performance. This interpretation is strengthened by the finding that the mean levels of ability tilt for the individuals in these generalist jobs closely mirrored the sample as a whole, indicating that the findings cannot be attributed to a restriction of range. More research is needed to determine what additional factors may influence whether ability tilt has positive or negative effects on performance in balanced or generalist jobs.

### 4.1. Theoretical Implications

The findings of this study have implications for research in the organizational sciences (e.g., industrial/organizational psychology and human resources management). Although these fields have begun to develop a renewed appreciation for specific cognitive abilities for predicting work-related criteria, existing research has given little attention to patterns among specific abilities. Thus, an important goal of this study was to contribute to a new line of research examining relations between cognitive ability patterns and various performance criteria. While prior research has found strong evidence that ability tilt influences individuals’ career choices and accomplishments, the present study suggests that tilt offers only a small contribution to the prediction of job performance. In this study, tilt provided relatively important increases in validity over *g* alone, but did not offer much incremental validity over job-relevant specific abilities. Thus, while the results of this study indirectly affirm the importance of specific abilities, the value of tilt is less clear. However, because the overall pattern of results is consistent with prior research, and there are theoretical reasons to expect that alignment between an individual’s specific abilities and job requirements may influence performance, these relationships may be worth investigating further in other samples. We acknowledge that the use of tilt scores has been subject to methodological critiques (Sorjonen et al. 2022); however, a growing body of research has shown that tilt provides unique insights (Coyle 2016, 2019, 2020; Coyle et al. 2014, 2015; Davison et al. 2014; Lubinski 2009; Lubinski et al. 2001; Park et al. 2008; Wai et al. 2018, 2022). In addition to further ability tilt research, it may be worth expanding this line of inquiry to more complex patterns of ability. A natural next step would be to assess how profiles comprised of multiple specific cognitive abilities relate to performance in specific jobs. This could be further extended by breaking down the criterion space into more discrete elements of performance. Such research would further our understanding of the role that specific abilities play in predicting job performance.

### 4.2. Practical Implications

The empirical findings of this study also have implications for personnel selection in organizations. Despite the small effect sizes observed, there are reasons to believe that ability tilt may, in some instances, be a useful predictor for jobs with similarly tilted requirements. While a predictor’s validity is a major determinant of its utility, practitioners must also consider a variety of nonstatistical factors, including cost (Lang et al. 2010). When deciding whether to add a predictor to an existing selection battery, then, one must weigh the relative improvement in prediction (i.e., incremental validity) against the additional costs incurred. In cases where a selection battery uses a cognitive ability test that includes measures of specific cognitive abilities, practitioners could easily compute tilt scores and incorporate them into the selection system at little to no additional cost. Thus, even if the incremental validity of tilt is small, it may offer utility as a no-cost improvement to the predictive validity an existing test.

One caveat is that ability tilt was found to relate positively to performance only in jobs with similarly tilted job requirements. Ability tilt bore no relation to performance in jobs that required various specific abilities to a similar degree, and thus would likely not be useful for predicting performance in jobs with more balanced cognitive requirements. This may seem to limit the usefulness of tilt as a predictor; however, it is worth noting that 58 of the 80 jobs (72.5%) examined in this study demonstrated tilted job requirements, and 42 jobs (52.5%) had tilted requirements between more than one pair of specific abilities. This suggests that measures of ability tilt would be of practical value for most jobs. Of course, validation would be required to ensure the use of tilt is appropriate to a given application, particularly given the inconsistences observed in this study. There may also be tradeoffs to consider in terms of fairness, as prior research has found gender differences in certain types of ability tilt, such as men showing greater math > verbal tilts and women showing greater verbal > math tilts (e.g., Coyle et al. 2015; Davison et al. 2014; Wai et al. 2018). Thus, implementing ability tilt into a selection system should be done carefully so as not to introduce gender-related adverse impacts.

### 4.3. Study Limitations

One limitation of this study is that the GATB was developed based on empirical methods (i.e., factor analyses) that were not guided by a specific theory or model of cognitive ability. This raises questions about the construct validity of the GATB aptitudes. Nevertheless, the aptitude dimensions defined by the GATB closely approximate specific ability factors included in contemporary multi-strata models of cognitive ability such as CHC. Additionally, care was taken to ensure that appropriate comparisons were made between the GATB aptitudes and the specific abilities in Fleishman’s taxonomy on which the O*NET job analysis ratings were based.

Another limitation concerns the nature of the GATB sample. This sample has previously been considered representative of U.S. workers, as evidenced by its use for validity generalization research (e.g., Hunter 1980; Hunter and Hunter 1984; Schmidt and Hunter 1998), which makes the findings of this study comparable to prior research. Nevertheless, certain jobs are notably under-represented in the sample (e.g., supervisory positions, STEM occupations, and other high-complexity jobs) and the data are now several decades old. Additionally, some have questioned the quality of the GATB data on the grounds that they were collected haphazardly over a period of almost two decades from a variety of samples (Hauser 2002). Steps were taken in the data cleaning process to address potential quality issues. Nevertheless, expanding ability tilt research to other samples more representative of today’s workforce may be a fruitful area of future inquiry and may yield more robust findings.

A third limitation of this study is the small samples for certain job tilts. Specifically, only one job required greater quantitative reasoning than written comprehension and only six jobs required greater quantitative reasoning than perceptual speed. These job groups were not as tilted as the other groups examined in the study and were not as diverse in terms of their composition; therefore, some caution is warranted in the interpretation of the results for these particular job groups. This concern is somewhat tempered by considering the overall pattern of tilt effects across all jobs examined in the study. However, future research should examine the effects of ability tilt on performance in jobs with a strong emphasis on quantitative reasoning.

## 5. Conclusions

This study contributes to the burgeoning literature on specific abilities in the workplace, by examining whether ability tilt between pairs of specific cognitive abilities differentially relates to job performance based on whether or not the tilt matches job requirements. Using GATB and performance data taken from a large sample of U.S. workers, it was found that, more often than not, ability tilt relates positively to performance when it aligns with job requirements and relates negatively to performance when it does not align with job requirements. While tilt contributed moderate increases in predictive validity over *g*, evidence of the incremental validity of tilt over specific abilities was limited. These results further our understanding of the complex relationship between cognitive ability and job performance and offer potential avenues for future research.

## Figures and Tables

**Table 1:** Composition of the GATB & Definitions of Aptitudes Measured by the GATB.

Aptitude	Definition	Test(s)
G—General Learning Ability	The ability to “catch on” or understand instructions and underlying principles; the ability to reason and make judgments. Closely related to doing well in school.	Part 3—Three-Dimensional Space Part 4—Vocabulary Part 6—Arithmetic Reason
V—Verbal Aptitude	The ability to understand the meaning of words and to use them effectively. The ability to comprehend language, to understand relationships between words and to understand meaning of whole sentences and paragraphs.	Part 4—Vocabulary
N—Numerical Aptitude	Ability to perform arithmetic operations quickly and accurately.	Part 2—Computation Part 6—Arithmetic Reason
S—Spatial Aptitude	Ability to think visually of geometric forms and to comprehend the two-dimensional representation of three-dimensional objects. The ability to recognize the relationships resulting from the movement of objects in space.	Part 3—Three-Dimensional Space
P—Form Perception	Ability to perceive pertinent detail in objects or in pictorial or graphic material. Ability to make visual comparisons and discriminations and see slight differences in shapes and shadings of figures and widths and lengths of lines.	Part 5—Tool Matching Part 7—Form Matching
Q—Clerical Perception	Ability to perceive pertinent detail in verbal or tabular material. Ability to observe differences in copy, to proofread words and numbers, and to avoid perceptual errors in arithmetic computation. A measure of speed of perception which is required in many industrial jobs even when the job does not have verbal or numerical content.	Part 1—Name Comparison
K—Motor Coordination	Ability to coordinate eyes and hands or fingers rapidly and accurately in making precise movements with speed. Ability to make a movement response accurately and swiftly.	Part 8—Mark Making
F—Finger Dexterity	Ability to move the fingers, and manipulate small objects with the fingers, rapidly or accurately.	Part 11—Assemble Part 12—Disassemble
M—Manual Dexterity	Ability to move the hands easily and skillfully. Ability to work with the hands in placing and turning motions.	Part 9—Place Part 10—Turn

Note. Adapted from (U.S. Department of Labor 1970). GATB = General Aptitude Test Battery.

**Table 2:** Demographic Characteristics of Sample.

				Race							
		Gender							Age (Years)		
Job Family	*n*	% Female	% Male	% Caucasian	% African American	% Hispanic/Latino	% Asian American	% Native American	*M*	*SD*	Mean Tenure (Years)
Architecture and engineering	601	3	97	76	15	5	3	1	36.1	10.6	10.7
Life, physical, and social science	95	20	80	92	-	6	1	1	31.9	9.7	7.8
Legal	594	99	1	77	15	6	2	<1	29.8	11.3	7.1
Healthcare practitioners and technical	1389	71	29	63	26	6	5	<1	30.9	9.1	5.8
Healthcare support	329	73	27	35	57	6	1	1	35.8	12.3	6.3
Protective service	849	20	80	46	46	7	<1	2	33.3	9.5	4.7
Food preparation and serving related	632	81	19	52	37	7	1	3	34.4	13.2	6.5
Personal care and service	528	90	10	67	20	8	2	3	26.9	9.5	1.9
Sales and related	409	86	14	63	27	6	2	2	35.3	14.4	6.2
Office and administrative support	5621	82	18	60	31	6	2	1	30.4	10.9	4.7
Construction and extraction	697	1	99	70	20	6	<1	4	32.1	9.9	8.2
Installation, maintenance, and repair	2740	1	99	71	17	8	1	2	35.5	10.5	10.7
Production	8316	41	59	57	32	8	1	2	32.9	10.6	6.3
Transportation and material moving	1194	69	31	62	30	5	1	2	36.8	12.0	8.4
Overall	23,994	51	49	61	29	7	1	2	32.6	11.0	6.5

Note. Tenure refers to total experience in current job, not just with current employer.

**Table 3:** Twenty Types of Ability Tilt Derived from Study Variables.

Verbal Tilt	Quantitative Tilt	Spatial Tilt	Form Perception Tilt	Clerical Perception Tilt
V > N	N > V	S > V	P > V	Q > V
V > S	N > S	S > N	P > N	Q > N
V > P	N > P	S > P	P > S	Q > S
V > Q	N > Q	S > Q	P > Q	Q > P

Note. Tilts between form perception and clerical perception (i.e., P > Q and Q > P) were excluded from the study since both correspond to O*NET ratings of perceptual speed.

**Table 4:** GATB Dimensions & Equivalent Cognitive Abilities from O*NET.

GATB Dimension	O*NET Ability
Verbal aptitude	Written comprehension
Numerical aptitude	Mathematical reasoning, number facility
Spatial aptitude	Visualization
Form perception	Perceptual speed
Clerical perception	Perceptual speed

**Table 5:** Sample Sizes and Sample Job Titles for Job Groups.

Tilt Type	*k*	*n*	Sample Job Titles
WC > QR	10	3375	Court reporters; file clerks; telephone operators; proofreaders and copy markers
QR > WC	1	287	Tellers
WC > Vz	16	5623	Court reporters; bookkeeping, accounting, and auditing clerks; proofreaders and copy markers
Vz > WC	17	5403	Sheet metal workers; automotive body and related repairers; welders, cutters, and welder fitters
WC > PS	16	4482	Proofreaders and copy markers; telephone operators; interviewers; cargo and freight agents
PS > WC	13	3974	Extruding, forming, pressing, and compacting machine setters, operators, and tenders; surgical technologists
QR > Vz	11	3464	Bookkeeping, accounting, and auditing clerks; tellers; general office clerks
Vz > QR	13	5126	Structural iron and steel workers; electrical and electronic equipment assemblers; general maintenance and repair workers
QR > PS	6	2100	Bookkeeping, accounting, and auditing clerks; tellers; cargo and freight agents
PS > QR	9	2847	Extruding, forming, pressing, and compacting machine setters, operators, and tenders; proofreaders and copy markers; tire builders
Vz > PS	16	4626	Hairdressers, hairstylists, and cosmetologists; structural metal fabricators and fitters; upholsterers
PS > Vz	9	3238	File clerks; proofreaders and copy markers; data entry keyers; court reporters

Note. WC = written comprehension; QR = quantitative reasoning; Vz = visualization; PS = perceptual speed. *k* refers to number of jobs and *n* refers to number of participants.

**Table 6:** Mean Levels of Ability Tilt Across All Jobs.

Tilt Type	*M*	*SD*
V > N	12.84	9.08
N > V	10.89	8.14
V > S	13.28	10.28
S > V	16.18	11.80
V > P	12.87	9.82
P > V	19.93	13.82
V > Q	7.97	6.61
Q > V	19.35	12.65
N > S	14.04	10.80
S > N	17.97	12.79
N > P	11.61	8.94
P > N	21.07	14.40
N > Q	7.83	6.51
Q > N	21.51	13.47
S > P	13.25	9.90
P > S	18.51	13.18
S > Q	12.94	9.91
Q > S	21.99	15.36

Note. Tilt is computed as the within-person score difference between two specific ability dimensions. V = verbal aptitude; N = numerical aptitude; S = spatial aptitude; P = form perception; Q = clerical perception. Mean levels of tilt were computed within the distribution of tilt scores greater than zero for each pair (e.g., mean V > N tilt is the mean of all V—N tilt scores greater than zero).

**Table 7:** Correlations between Ability Tilt and Job Performance in Jobs with Matching Requirements, Controlling for *g*.

Tilt Type	*k*	*n*	*r*
V > N	10	3375	.005
N > V	1	287	.038
V > S	16	5623	.106 ***
S > V	17	5403	.019
V > P	16	4482	.120 ***
P > V	13	3974	−.002
V > Q	16	4482	.051 ***
Q > V	13	3974	.027
N > S	11	3464	.090 ***
S > N	13	5126	−.028 *
N > P	6	2100	.046 *
P > N	9	2847	−.020
N > Q	6	2100	.051 *
Q > N	9	2847	−.020
S > P	16	4626	.071 ***
P > S	9	3238	.044 *
S > Q	16	4626	.054 ***
Q > S	9	3238	.083 ***

Note. Tilt is computed as the within-person score difference between two specific ability dimensions. V = verbal aptitude; N = numerical aptitude; S = spatial aptitude; P = form perception; Q = clerical perception. Correlations between tilt scores and the job performance criterion were computed controlling for *g*. * *p* < .05. ** *p* < .01. *** *p* < .001.

**Table 8:** Correlations between Ability Tilt and Job Performance in Jobs with Mismatched Requirements, Controlling for *g*.

Tilt Type	*k*	*n*	*r*
V > N	1	287	−.038
N > V	79	23,707	.025 ***
V > S	37	10,220	−.029 **
S > V	43	13,774	−.057 ***
V > P	24	7477	.015
P > V	55	16,212	−.056 ***
V > Q	24	7477	−.012
Q > V	55	16,212	−.019 *
N > S	63	18,993	.045 ***
S > N	16	4855	−.078 ***
N > P	73	21,740	.065 ***
P > N	6	2100	−.046 *
N > Q	73	21,740	.029 ***
Q > N	6	2100	−.051 *
S > P	45	14,239	−.020 *
P > S	33	9316	−.071 ***
S > Q	45	14,239	−.060 ***
Q > S	33	9316	−.049 ***

Note. Tilt is computed as the within-person score difference between two specific ability dimensions. V = verbal aptitude; N = numerical aptitude; S = spatial aptitude; P = form perception; Q = clerical perception. Correlations between tilt scores and the job performance criterion were computed controlling for *g*. * *p* < .05. ** *p* < .01. *** *p* < .001.

**Table 9:** Relative Weights and Incremental Validity Analyses for g, Specific Abilities, and Ability Tilt in Jobs Requiring Tilt.

		*g*	Specific Ability	Ability Tilt			Incremental Validities				
Tilt Type	*n*	ε_j_ (%)	ε_j_ (%)	ε_j_ (%)	*R* ^2^	*R_g_* ^2^	Δ*R*^2^	% Increase in *R*^2^	*R_g_*_s_^2^	Δ*R*^2^	% Increase in *R*^2^
V > N	3375	.039 (43.7)	.042 (46.5)	.009 (9.8)	.090	.077	.013	17%	.083	.006	7%
N > V	287	.020 (35.6)	.032 (56.9)	.004 (7.5)	.057	.040	.017	43%	.056	.000	0%
V > S	5623	.053 (62.3)	.026 (30.2)	.006 (7.6)	.085	.075	.010	13%	.079	.006	8%
S > V	5403	.015 (55.2)	.010 (37.3)	.002 (7.5)	.028	.026	.002	8%	.026	.002	8%
V > P	4482	.047 (57.8)	.028 (34.9)	.006 (7.3)	.081	.069	.012	17%	.075	.006	8%
P > V	3974	.021 (72.6)	.007 (24.7)	.001 (2.8)	.028	.026	.002	8%	.028	.001	4%
V > Q	4482	.040 (53.2)	.033 (43.9)	.002 (2.9)	.075	.069	.007	10%	.075	.000	0%
Q > V	3974	.017 (61.8)	.009 (33.3)	.001 (4.9)	.027	.026	.001	4%	.027	.000	0%
N > S	3464	.036 (51.1)	.026 (36.5)	.009 (12.5)	.071	.059	.013	22%	.068	.004	6%
S > N	5126	.014 (46.6)	.011 (35.7)	.005 (17.7)	.031	.027	.004	15%	.027	.004	15%
N > P	2100	.022 (46.4)	.023 (48.7)	.002 (4.9)	.047	.040	.007	18%	.047	.000	0%
P > N	2847	.019 (61.1)	.011 (34.2)	.001 (4.7)	.032	.031	.000	0%	.032	.000	0%
N > Q	2100	.023 (48.4)	.021 (44.3)	.003 (7.3)	.048	.040	.008	20%	.047	.000	0%
Q > N	2847	.018 (55.3)	.012 (37.7)	.002 (6.9)	.032	.031	.000	0%	.032	.000	0%
S > P	4626	.026 (68.7)	.009 (24.0)	.003 (7.2)	.038	.032	.006	19%	.033	.005	15%
P > S	3238	.069 (71.6)	.022 (22.7)	.005 (5.7)	.097	.087	.010	11%	.089	.008	9%
S > Q	4626	.023 (69.0)	.009 (26.1)	.002 (4.9)	.034	.032	.001	3%	.033	.001	3%
Q > S	3238	.065 (68.1)	.024 (25.5)	.006 (6.4)	.095	.087	.008	9%	.087	.007	8%

Note. Tilt is computed as the within-person score difference between two specific ability dimensions. Specific ability in each model is the primary (dominant) ability in the tilt. V = verbal aptitude; N = numerical aptitude; S = spatial aptitude; P = form perception; Q = clerical perception. ε_j_ = relative weight. *R_g_*^2^ = total variance explained by *g. R_g_*_s_^2^ = total variance explained by *g* and specific abilities. While we did not present a hypothesis for jobs with balanced ability requirements (RQ1), based on cognitive expertise theory (Connell et al. 2003), we suspected that ability tilt would relate negatively to performance in these jobs. Results did not support this expectation (see Table 10). Only one of the eighteen types of ability tilt (Q > S tilt) related negatively to performance in jobs with balanced ability requirements; two types of tilt (V > S tilt and P > V tilt) related positively to performance in these jobs, and the other fifteen types of tilt were not significantly related to performance in these jobs.

**Table 10:** Correlations between Ability Tilt and Job Performance in Jobs with Balanced Ability Requirements.

Tilt Type	*k*	*n*	Ability Tilt
V > N	9	1449	.041
N > V	9	1064	.020
V > S	9	1105	.063 *
S > V	9	1412	.004
V > P	9	812	.060
P > V	9	1682	.064 **
V > Q	9	414	−.034
Q > V	9	2091	−.020
N > S	9	904	−.024
S > N	9	1595	−.004
N > P	9	665	.002
P > N	9	1836	.024
N > Q	9	327	−.100
Q > N	9	2169	−.033
S > P	9	953	.045
P > S	9	1550	.020
S > Q	9	698	−.063
Q > S	9	1817	−.053 *

Note. Tilt is computed as the score difference in two specific ability dimensions. V = verbal aptitude; N = numerical aptitude; S = spatial aptitude; P = form perception; Q = clerical perception. Correlations between tilt scores and the job performance criterion were computed controlling for *g*. * *p* < .05. ** *p* < .01.

## Data Availability

The GATB data used in this study are maintained by the National Center for O*NET Development. Requests for the GATB data can be sent to: onet@onetcenter.org.

## Appendix A

The data reported in this manuscript were obtained from publicly available data shared by the National Center for O*NET Development and the U.S. Department of Labor (https://www.onetonline.org, accessed on 14 December 2018). The data used in this study were accessed through the National Center for O*NET Development on 14 December 2018. The dataset provided by O*NET contains all General Aptitude Test Battery (GATB) data collected through the 1980s. A large number of published articles have used these data over the years. To our knowledge, these data have been used in the meta-analytic work of Schmidt and Hunter (e.g., Hunter 1980; Hunter and Hunter 1984; Schmidt and Hunter 1998) as well as a more recent paper by McDaniel and Kepes (2014; see full citations below). The variables and relationships examined in the present article have not been examined in any previous or current articles, nor to the best of our knowledge in any papers that will be under review soon.
